# Beyond inflammation: metabolic implications of biological and TsDMARD therapies in dermatologic and rheumatologic diseases

**DOI:** 10.3389/fimmu.2026.1668159

**Published:** 2026-02-12

**Authors:** Salvatore Corrao, Salvatore Scibetta, Nicola Pardo, Ignazio Cangemi, Luigi Mirarchi, Giacomo Corrao, Simona Amodeo, Luigi Calvo

**Affiliations:** 1 Department of Clinical Medicine, Internal Medicine Unit with rheumatology, dermatology, diabetology and tertiary diabetic foot healthcare, National Relevance and High Specialization Hospital Trust ARNAS Civico, Palermo, Italy; 2 Department of Health Promotion Sciences, Maternal and Infant Care, Internal Medicine and Medical Specialties [PROMISE], University of Palermo, Palermo, Italy; 3 Institute for Biomedical Research and Innovation (IRIB), National Research Council (CNR), Palermo, Italy; 4 Department of Internal Medicine, Azienda Ospedaliera Universitaria “Policlinico G. Martino, University of Messina, Messina, Italy

**Keywords:** biologic DMARDs, immunometabolism, inflammation, insulin resistance, precision medicine

## Abstract

Biologic and targeted synthetic disease-modifying antirheumatic drugs (DMARDs) have transformed the management of chronic inflammatory diseases. Yet their therapeutic impact extends beyond cytokine suppression, influencing systemic metabolic pathways that are increasingly recognised as central to immune regulation. This narrative review examines the immunometabolic effects of major biologic and targeted synthetic DMARD classes used in dermatologic and rheumatologic diseases. We synthesise evidence on how these agents modulate insulin sensitivity, lipid metabolism, adipokine profiles, mitochondrial function, and adipose-tissue inflammation thereby shaping cardiovascular and metabolic risk. TNF inhibitors show heterogeneous metabolic effects, whereas IL-6 blockade and JAK inhibition consistently improve glycemic parameters despite inducing characteristic lipid changes. IL-17 and IL-23 inhibitors may attenuate adipose inflammation, while TYK2 inhibitors appear metabolically neutral. Through integration of mechanistic insights and clinical data, this review highlights the need to incorporate metabolic phenotyping into therapeutic decision-making. Understanding the distinct metabolic fingerprints of DMARDs may enable more precise patient stratification and support emerging combinatorial strategies with metabolic agents such as GLP-1 receptor agonists and SGLT2 inhibitors. These perspectives underscore the translational importance of viewing DMARD therapies not only as immunomodulators but also as systemic metabolic regulators.

## Introduction

Chronic immune-mediated inflammatory diseases (IMIDs) such as psoriasis, psoriatic arthritis, rheumatoid arthritis, and spondyloarthritis have traditionally been conceptualised through the lens of aberrant immune activation. However, growing evidence demonstrates that these conditions exist at the intersection of inflammation and metabolism. Systemic metabolic dysfunction including insulin resistance, visceral adiposity, mitochondrial stress, and dyslipidemia—does not merely accompany IMIDs (1, 2); it actively shapes immune pathways, disease activity, and therapeutic response (3).

This bidirectional relationship defines the field of immunometabolism, in which immune cell activation, cytokine signalling, and tissue inflammation are tightly coupled to cellular energetics, nutrient utilisation, and mitochondrial biology. Importantly, immune signalling pathways targeted by biologic and targeted synthetic DMARDs, such as TNF-α, IL-6, IL-17-23, and JAK–STAT cascades, also regulate key metabolic circuits. This overlap suggests that immunomodulatory therapies may exert clinically meaningful metabolic effects, with implications for cardiometabolic risk and treatment selection (4). Similarly, metabolic organs such as the liver, adipose tissue, and skeletal muscle express immune sensors and participate actively in inflammatory signalling, particularly in chronic low-grade inflammation characteristic of metabolic syndrome (5). This overlap is particularly evident in psoriatic disease and RA, where elevated levels of tumour necrosis factor-alpha (TNF-α), interleukin-6 (IL-6), and interleukin-17 (IL-17) not only perpetuate immune dysfunction but also interfere with glucose homeostasis and lipid metabolism (6, 7).

Therapies initially developed to block cytokine-driven inflammation such as biologic DMARDs (bDMARDs) and targeted synthetic DMARDs (tsDMARDs) now emerge as tools that may modulate systemic metabolism. For example, anti-TNF agents have shown variable but sometimes favourable effects on insulin sensitivity and endothelial function (8), while IL-17 and IL-23 inhibitors may indirectly affect adipose tissue inflammation and body composition (9). Moreover, Janus kinase (JAK) inhibitors influence lipid metabolism, adipokine secretion, and mitochondrial pathways, suggesting they might simultaneously reprogram immune and metabolic pathways (10). Though still under investigation, these observations have opened a critical question: can anti-inflammatory therapies also serve as metabolic modulators?

This question is clinically urgent. Individuals with IMIDs frequently bear a significant burden of cardiometabolic comorbidities, including type 2 diabetes, non-alcoholic fatty liver disease (NAFLD), dyslipidemia, and increased cardiovascular mortality. For instance, in patients with psoriasis, the prevalence of metabolic syndrome is nearly twice that of the general population (11). In RA, systemic inflammation independently contributes to insulin resistance and subclinical atherosclerosis, regardless of body mass index (12). Failing to address these interconnected pathways may lead to residual cardiovascular risk despite successful control of articular or dermatologic symptoms.

In this context, the immunometabolic perspective is theoretical and has therapeutic and prognostic consequences. The emergence of this cross-disciplinary framework urges clinicians and researchers to go “beyond inflammation” and assess the metabolic implications of immunomodulatory therapies more rigorously. Furthermore, it necessitates a more personalised approach, where therapeutic decisions are informed by disease phenotype and the patient’s metabolic profile. Despite this emerging evidence, the metabolic consequences of DMARD therapy remain under-recognised in routine practice and inadequately integrated into treatment algorithms. This review addresses this translational gap by examining how therapeutic classes with distinct mechanisms of action modulate systemic metabolic pathways. We selected biologic and targeted synthetic DMARDs based on their widespread clinical use and mechanistic relevance to immunometabolic regulation. Our objective is to provide clinicians and researchers with an updated synthesis of metabolic fingerprints across DMARD classes, illustrating how these effects may inform precision medicine approaches and patient stratification.

## Pathophysiological intersections: immunological and metabolic mechanisms

The immune system and metabolic networks operate as deeply interconnected systems. Immune activation requires rapid metabolic reprogramming, while metabolic stress amplifies inflammatory signalling. Understanding this bidirectional relationship is essential to contextualise how DMARDs reshape both immune and metabolic pathways.

## Immune cell metabolic reprogramming

Activation of T cells, macrophages, and dendritic cells induces a shift from oxidative phosphorylation to aerobic glycolysis, supporting proliferation, cytokine production, and biosynthesis. Naïve T cells rely predominantly on oxidative phosphorylation (OXPHOS), but once activated, they switch to aerobic glycolysis, a phenomenon reminiscent of the Warburg effect observed in cancer cells (13). This metabolic shift facilitates rapid ATP production, nucleic acid synthesis, and membrane biogenesis, which are critical for proliferation and cytokine production. Similarly, M1 macrophages, which drive pro-inflammatory responses, adopt a glycolytic profile, while M2 macrophages, involved in tissue repair and resolution, favour fatty acid oxidation and mitochondrial respiration (14).

These metabolic programs are not passive consequences of activation; they are integral to immune function. For instance, glycolysis sustains IFN-γ production in Th1 cells and IL-17 in Th17 cells, while inhibition of glycolysis can selectively dampen inflammatory responses without impairing anti-inflammatory pathways (15). Moreover, this metabolic transition is orchestrated by HIF-1α, mTOR, and NF-κB pathways, while regulatory T cells preferentially rely on fatty acid oxidation and mitochondrial respiration. Key intermediates such as succinate, citrate, and itaconate act as immunometabolic signalling molecules that sustain inflammatory phenotypes (16).

## Systemic inflammation and insulin resistance

Chronic inflammation in rheumatoid arthritis and psoriasis contributes to insulin resistance through multiple mechanisms. Pro-inflammatory cytokines, such as TNF-α, IL-1β, and IL-6, interfere with insulin signalling pathways in adipose tissue, the liver, and skeletal muscle (17). TNF-α induces the serine phosphorylation of IRS-1, reducing insulin receptor signalling, while IL-6 promotes hepatic gluconeogenesis and lipid accumulation (18, 19).

Mechanistically, TNF-α impairs insulin action by activating stress and inflammatory kinases particularly JNK and IKKβ thereby promoting inhibitory serine phosphorylation of IRS-1 and blunting the insulin receptor–IRS-1–PI3K–AKT cascade in adipose tissue and skeletal muscle (20). This results in reduced GLUT4 translocation, diminished glucose uptake, and a feed-forward amplification of lipolysis and ectopic lipid deposition. Conversely, IL-6 signaling through the IL-6R/gp130 complex activates JAK-STAT3 in hepatocytes, upregulating gluconeogenic programs (including PEPCK and glucose-6-phosphatase) and contributing to increased hepatic glucose production (21); IL-6 can additionally modulate SOCS proteins, further dampening insulin receptor signaling.

The resulting metabolic derangements hyperglycemia, dyslipidemia, and visceral adiposity create a vicious cycle that exacerbates immune activation, particularly in obese individuals, where adipose tissue reservoirs pro-inflammatory cytokines (22).

Adipose tissue is a metabolically active immune organ. In obesity and metabolic syndrome, it becomes infiltrated by macrophages, dendritic cells, and even CD8+ T cells, which produce chemokines and cytokines that propagate local and systemic inflammation (23). In this context, immune cells not only respond to metabolic stress—they create it, establishing a state of chronic low-grade inflammation termed “metaflammation” (24).

## Mitochondrial dysfunction and redox stress

Mitochondria act as hubs coupling metabolism to innate immunity. In IMIDs, mitochondrial dysfunction promotes reactive oxygen species (ROS) overproduction, activates inflammasomes, and triggers NF-κB signalling. Mitochondrial dysfunction, characterised by impaired oxidative phosphorylation and increased ROS, is a hallmark of autoimmune and inflammatory diseases. In synovial tissue from RA patients, mitochondrial ROS activate NF-κB and inflammasome pathways, enhancing the production of IL-1β and IL-18 (25). Similarly, in psoriatic skin, oxidative stress drives keratinocyte hyperproliferation and cytokine release, perpetuating the inflammatory loop (26). Release of mitochondrial DNA functions as a damage-associated molecular pattern (DAMP), amplifying innate immune responses through toll-like receptor 9 (TLR9) and cyclic GMP-AMP synthase (cGAS)–STING activation (27). Thus, these mechanisms contribute to persistent inflammation in joints, skin, adipose tissue, and vasculature.

## Adipose tissue as an immunometabolic organ

Visceral adipose tissue in IMIDs is infiltrated by macrophages, T cells, and dendritic cells that drive chronic low-grade inflammation (“metaflammation”). Cytokines such as TNF-α and IL-6 impair insulin signalling by promoting IRS-1 serine phosphorylation, increasing lipolysis, and stimulating hepatic gluconeogenesis. Altered adipokine secretion including increased leptin and decreased adiponectin further couples’ metabolic dysfunction to immune dysregulation.

### Systemic crosstalk across liver, muscle, and skin

Each tissue microenvironment uniquely modulates the immune-metabolic crosstalk. The liver integrates inflammatory and metabolic cues, contributing to NAFLD/MASH in IMIDs (28). In psoriatic skin, keratinocytes adopt glycolytic and pro-oxidative profiles that perpetuate inflammation (29).

In the joints, synovial fibroblasts in RA display a “transformed” phenotype characterised by hypermetabolism, increased glycolytic flux, and resistance to apoptosis. These aggressive synoviocytes perpetuate inflammation and joint destruction, and recent work suggests that modulating their metabolic program may reverse pathogenic behaviour (30). Skeletal muscle undergoes catabolic remodelling via IL-6 and TNF-α signalling.

These tissue-specific responses underscore the systemic nature of immunometabolic dysfunction. This framework contextualises how DMARDs, by modulating cytokine networks and intracellular signalling pathways, may also recalibrate metabolic homeostasis.

**Figure 1:**
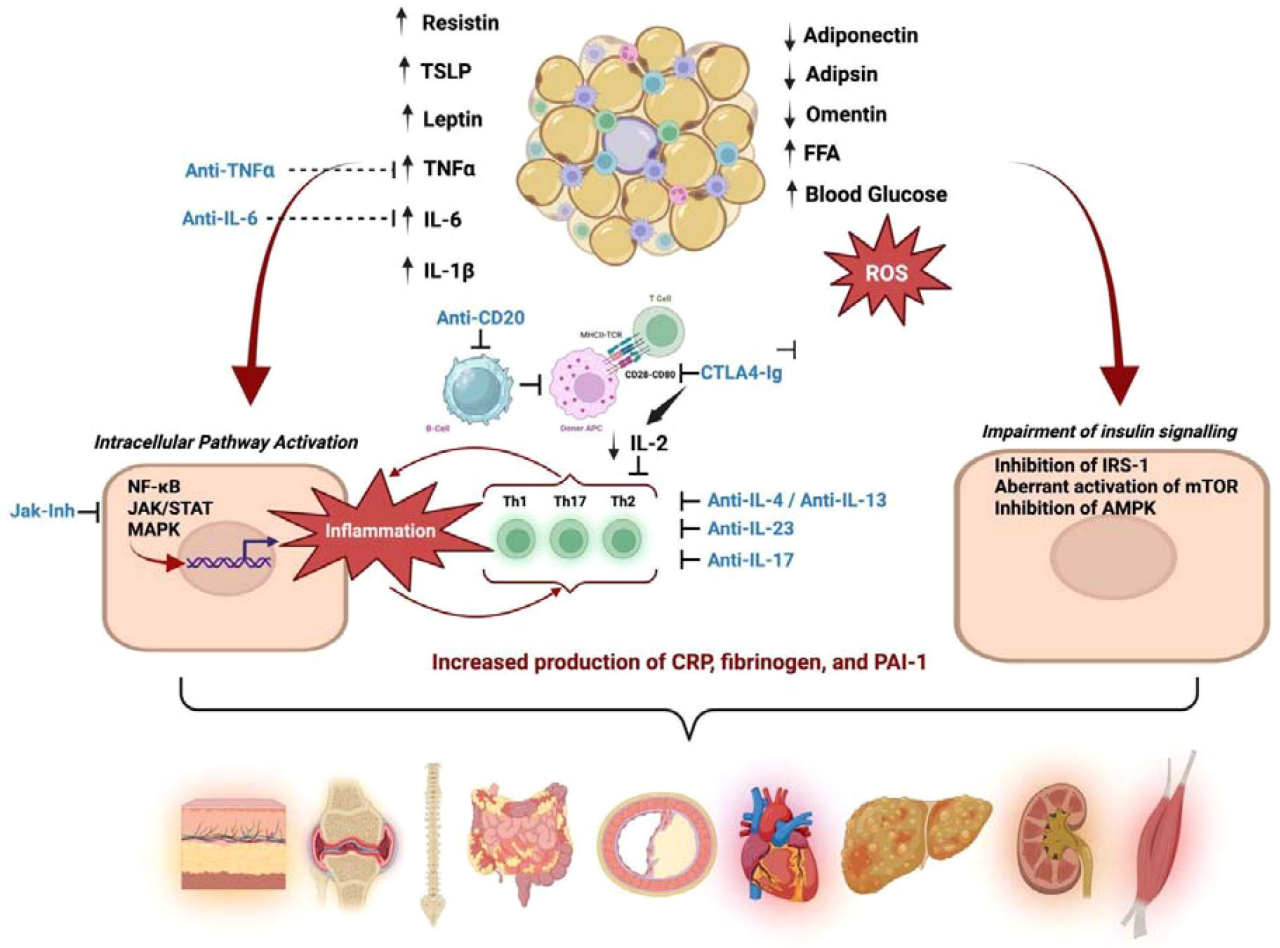
Targeting the immuno-metabolic crosstalk linking adipose tissue dysfunction to insulin resistance and systemic organ damage. AMPK, AMP-activated protein kinase; APC, antigen-presenting cell; CRP, C-reactive protein; CTLA4-Ig, cytotoxic T-lymphocyte antigen-4 immunoglobulin fusion protein; FFA, free fatty acids; IL, interleukin; IRS-1, insulin receptor substrate-1; JAK, Janus kinase; MAPK, mitogen-activated protein kinase; mTOR, mammalian target of rapamycin; NF-κB, nuclear factor-κB; PAI-1, plasminogen activator inhibitor-1; ROS, reactive oxygen species; STAT, signal transducer and activator of transcription; TCR, T-cell receptor; Th, T helper; TNF-α, tumor necrosis factor-α; TSLP, thymic stromal lymphopoietin.

As shown in **Figure 1**, dysfunctional adipose tissue acts as an immunometabolic hub by increasing pro-inflammatory adipokines and cytokines (e.g., leptin, resistin, TSLP, TNF-α, IL-6, IL-1β) while reducing protective mediators such as adiponectin, adipsin, and omentin. The concomitant rise in free fatty acids, hyperglycemia, and ROS activates intracellular inflammatory pathways (NF-κB, JAK/STAT, MAPK) and sustains adaptive immune responses (Th1/Th17/Th2), leading to higher systemic inflammatory and pro-thrombotic markers (CRP, fibrinogen, PAI-1). In parallel, these inflammatory cues impair insulin signaling via IRS-1 inhibition, aberrant mTOR activation, and AMPK suppression, thereby promoting insulin resistance and multi-organ comorbidity.

### Biologics and metabolism: more than disease control

Biologic disease-modifying antirheumatic drugs (bDMARDs) have dramatically reshaped the therapeutic landscape of immune-mediated inflammatory diseases (IMIDs), achieving unprecedented control of disease activity in conditions such as rheumatoid arthritis, psoriatic arthritis, psoriasis, and ankylosing spondylitis. Developed initially to interrupt inflammatory cascades notably by neutralising cytokines such as TNF-α, IL-6, or IL-17 biologics are now recognised for their broader systemic effects, including metabolism. The increasing prevalence of metabolic comorbidities among patients with IMIDs has prompted a renewed interest in the “off-target” or “secondary” metabolic actions of these agents (31). Though sometimes subtle or heterogeneous, these effects hold significant clinical relevance in the context of cardiovascular risk, insulin resistance, and adipose tissue function.

### Anti-TNF agents: heterogeneous and context-dependent effects

Among biologics, TNF-α inhibitors are the most extensively studied in terms of their metabolic consequences. TNF-α drives insulin resistance, adipose inflammation, and endothelial dysfunction. TNF-α plays a pivotal role in insulin resistance, by neutralising TNF-α, agents such as infliximab, adalimumab, and etanercept might theoretically improve insulin sensitivity and metabolic outcomes. Several clinical studies have supported this hypothesis, demonstrating improved fasting glucose levels, insulin resistance indices, and lipid profiles in patients with RA or psoriasis who received anti-TNF therapies (32, 33).

However, metabolic responses are inconsistent across studies. Variability likely reflects differences in obesity status, disease duration, and TNF burden in adipose tissue (34). These discrepancies may be due to differences in patient populations, disease duration, baseline metabolic status, or the specific anti-TNF agent used. It is also increasingly apparent that the anti-inflammatory effect alone may be insufficient to reverse entrenched metabolic dysfunction, particularly in individuals with long-standing obesity or established type 2 diabetes.Some patients experience weight gain or altered fat distribution, suggesting that anti-TNF agents may alleviate catabolic inflammation but not fully reverse underlying metabolic dysfunction.

### IL-17 and IL-23 inhibitors: metabolic modulation through adipose inflammation

Inhibitors of IL-17A (such as secukinumab and ixekizumab), IL 17A-17F (bimekizumab), and IL-23p19 (such as guselkumab, risankizumab, and tildrakizumab) have become high effectiveness therapies for moderate-to-severe psoriasis and are increasingly employed in the treatment of psoriatic arthritis. IL-17 is a key effector cytokine in psoriatic lesions, exerting direct effects on adipose tissue, liver, and endothelium. Experimental models suggest that IL-17 and IL-23 contribute to adipocyte dysfunction, endothelial stress, and NAFLD progression (35). Their inhibition may reduce adipose-tissue inflammation, improve adipokine balance, and attenuate Th17-driven metabolic stress.

From a mechanistic standpoint, IL-17 signals through IL-17RA/IL-17RC complexes expressed not only on immune cells but also on non-hematopoietic targets, including adipocytes and adipose stromal cells, thereby promoting chemokine production (e.g., CCL2 and CXCL family members) (36), myeloid recruitment, and amplification of local inflammatory tone. In contrast, IL-23 primarily acts upstream on IL-23R–expressing immune subsets classically Th17 cells and innate-like T cells (37) stabilizing and expanding IL-17–producing programs rather than signaling directly on adipocytes. Adipose tissue contains IL-17–producing lymphocytes, including Th17 and γδ T cells, which can participate in metaflammation and contribute to insulin resistance (38) through macrophage activation and adipokine dysregulation; thus, IL-23/IL-17 pathway blockade may attenuate adipose immune activation and improve immunometabolic homeostasis.

Early clinical evidence suggests improvements in insulin resistance markers and adipokine profiles, though long-term cardiometabolic implications require further investigation (39). For example, IL-23 blockade has been associated with reductions in C-reactive protein, leptin, and resistin, suggesting favourable shifts in adipokine profiles (40). Nonetheless, large-scale longitudinal studies are still needed to assess whether these changes translate into reductions in cardiovascular events or diabetes incidence.

### IL-6 inhibition: consistent glycemic benefits with characteristic lipid changes

IL-6 inhibitors such as tocilizumab improve insulin sensitivity and reduce HbA1c independently of inflammation control. These benefits reflect interruption of IL-6–driven hepatic gluconeogenesis, lipolysis, and muscle catabolism (41). Tocilizumab also has demonstrated significant efficacy in rheumatoid arthritis (RA) and giant cell arteritis. Importantly, studies have shown that IL-6 blockade can improve insulin sensitivity and reduce HbA1c levels in patients with RA, independent of changes in disease activity (42). This supports the notion that targeting IL-6 may have direct metabolic benefits due to the suppression of inflammation and the interruption of metabolic signalling pathways.

However, IL-6 blockade consistently raises LDL and HDL cholesterol (43). Despite these quantitative increases, the LDL/HDL ratio often remains stable, and markers of vascular inflammation improve, raising the possibility that lipid changes may be mechanistically distinct from atherosclerotic risk.

### Other biologic agents: CTLA4-Ig and anti-CD20 - selective and emerging metabolic actions

Abatacept, a fusion protein targeting the CD80/86:CD28 costimulatory axis, modulates T-cell activation and has shown efficacy in RA. Abatacept may enhance insulin sensitivity by restoring Treg-mediated metabolic homeostasis within adipose tissue (44). Similarly, rituximab, a CD20+ B cell-depleting antibody, exerts modest and variable metabolic effects, likely reflecting indirect reductions in inflammatory burden rather than direct immunometabolic modulation (45).

Abatacept (CTLA4-Ig) inhibits CD80/86–CD28 co-stimulation, thereby reducing effector T-cell activation and favoring a regulatory immune milieu. In adipose tissue, Tregs contribute to metabolic homeostasis by constraining local inflammation partly through IL-10 and TGF-β–linked programs (46, 47) and by limiting pro-inflammatory macrophage polarization and adipokine derangements that impair insulin signaling. Clinically, abatacept has been associated with improvements in insulin resistance indices in inflammatory arthritis (48), supporting a systemic immunometabolic effect; however, direct human data demonstrating increased adipose-resident Treg abundance or functional reprogramming under abatacept remain limited. We therefore frame Treg-mediated adipose immune normalization as a plausible mechanistic bridge that is consistent with co-stimulation biology and with observed metabolic improvements, while acknowledging the need for dedicated tissue-level studies.

These findings highlight the heterogeneity of immunometabolic responses to biologic therapy. Some effects may be class-specific, while others reflect shared consequences of systemic inflammation resolution. Importantly, the metabolic signature of each biologic may help inform therapeutic choice, particularly in patients with coexisting metabolic disease.

**Table 1** provides an overview of the metabolic effects associated with different biologic therapies.

**Table 1:** Biologics and tsDMARDs by class and indications.

Drug class	Molecule	Approved indications	Metabolic effects
Anti-TNFα	Adalimumab	RA, PsA, AS, PsO, IBD	Variable effects on lipids and insulin resistance
Anti-TNFα	Etanercept	RA, PsA, AS, PsO	Less lipid impact than other TNFi
Anti-TNFα	Infliximab	RA, PsA, AS, PsO, IBD	Possible HDL increase; variable data
Anti-TNFα	Certolizumab pegol	RA, PsA, AS, PsO	Limited metabolic data
Anti-TNFα	Golimumab	RA, PsA, AS, UC	Similar profile to other TNFi
Anti-IL-6R	Tocilizumab	RA, sJIA, GCA	↑ LDL & HDL; LDL/HDL ratio stable
Anti-IL-6R	Sarilumab	RA	Similar to tocilizumab
Anti-IL-17A	Secukinumab	PsA, PsO, AS	Limited data; potential adipose benefit
Anti-IL-17A	Ixekizumab	PsA, PsO, AS	Similar to secukinumab
Anti-IL-17A/F	Bimekizumab	PsA, PsO, AS	Dual IL-17 inhibition; promising adipose effects
Anti-IL-23	Guselkumab	PsA, PsO	Limited data; likely neutral
Anti-IL-23	Risankizumab	PsA, PsO	Similar to guselkumab
Anti-IL-23	Tildrakizumab	PsO	No metabolic data
Anti-IL-12/23	Ustekinumab	PsA, PsO, CD	Likely neutral
JAK Inhibitor	Tofacitinib	RA, PsA, UC	↑ LDL & HDL
JAK Inhibitor	Baricitinib	RA, AD	↑ LDL & HDL
JAK Inhibitor	Upadacitinib	RA, PsA, AD	↑ LDL & HDL
TYK2 Inhibitor	Deucravacitinib	PsO	Limited data; likely neutral
CTLA4-Ig	Abatacept	RA, PsA	Improves insulin resistance; ↑ lipids
Anti-CD20	Rituximab	RA, GPA, MPA	Variable effects on lipids and insulin resistance

AD, Atopic Dermatitis; AS, Ankylosing Spondylitis; CD, Crohn’s Disease; GCA, Giant Cell Arteritis; GPA, Granulomatosis with Polyangiitis; IBD, Inflammatory Bowel Disease; MPA, Microscopic Polyangiitis; PsA, Psoriatic Arthritis; PsO, Psoriasis; RA, Rheumatoid Arthritis; sJIA, Systemic Juvenile Idiopathic Arthritis; UC, Ulcerative Colitis.

### tsDMARDs and metabolic adaptation

The development of targeted synthetic disease-modifying antirheumatic drugs (tsDMARDs), most notably Janus kinase (JAK) inhibitors, has revolutionised the therapeutic armamentarium for immune-mediated inflammatory diseases (IMIDs). These small molecules act intracellularly, modulating the signalling pathways of multiple cytokines by interfering with the JAK-STAT (Signal Transducer and Activator of Transcription) cascade. Unlike monoclonal antibodies that bind extracellular cytokines or cell-surface receptors, tsDMARDs exert broader, pleiotropic effects due to their intracellular mechanism, affecting a wide range of immune and non-immune cells.

Beyond their efficacy in controlling inflammation in diseases such as rheumatoid arthritis (RA), psoriatic arthritis (PsA), and atopic dermatitis, increasing attention has turned to their metabolic consequences, which may complement or complicate their therapeutic action. These agents influence lipid metabolism, insulin sensitivity, mitochondrial function, and body composition, indicating that their immunologic and metabolic effects are deeply intertwined.

### JAK inhibitors: broad intracellular effects on metabolism

JAK inhibitors reshape multiple cytokine pathways relevant to metabolic regulation. One of the most consistently reported metabolic effects of JAK inhibitors is their impact on serum lipid profiles. Agents such as tofacitinib, baricitinib, upadacitinib, and filgotinib have been shown to consistently increase low-density lipoprotein (LDL) and high-density lipoprotein (HDL) cholesterol likely reflecting reversal of inflammation-driven lipid suppression rather than new-onset dyslipidemia (49). This lipid rise is thought to reflect the normalisation of lipid metabolism, which was previously suppressed by high-grade systemic inflammation, a phenomenon known as the lipid paradox in RA, where low cholesterol levels are paradoxically associated with heightened cardiovascular risk due to inflammation-driven catabolism (50).

The clinical implications of these lipid changes remain a topic of debate. While the numerical rise in LDL may appear concerning, the LDL/HDL ratio often remains stable or improves, and no definitive increase in major adverse cardiovascular events (MACE) has been demonstrated in large trials to date (51). Nonetheless, the U.S. FDA and EMA have issued warnings regarding increased cardiovascular and thromboembolic risks in specific populations receiving tofacitinib, underscoring the need for metabolic vigilance, particularly in patients with preexisting risk factors (52).

An important clinical nuance is the coexistence of seemingly favourable metabolic signals (modest improvements in insulin sensitivity or glycemic indices and reversal of inflammation-suppressed lipid levels) with regulatory warnings regarding increased cardiovascular and thromboembolic risk for some JAK inhibitors in selected populations (53). This apparent contradiction can be reconciled by recognizing that (i) lipid elevations under JAK inhibition often reflect normalization of the ‘lipid paradox’ observed in chronic inflammation rising LDL and HDL with relatively stable ratios—rather than a straightforward pro-atherogenic shift; yet (ii) hard safety outcomes (MACE/VTE) may be influenced by factors not captured by standard metabolic biomarkers, including patient risk enrichment (older age, smoking, prior CV disease), drug selectivity/dose, endothelial–hemostatic effects, and residual inflammatory–thrombotic pathways (53, 54). Accordingly, metabolic improvement does not necessarily translate into reduced cardiovascular event risk. We therefore underscore the need for careful baseline CV risk stratification, vigilant monitoring (including lipids and global risk), and proactive LDL-cholesterol management when JAK inhibitors are prescribed, particularly in high-risk individuals (54).

### JAK inhibitors and insulin sensitivity

Data on the effects of JAK inhibitors on insulin sensitivity and glucose metabolism are still emerging. Preclinical models have shown that JAK-STAT signaling regulates insulin receptor pathways and adipocyte differentiation, with JAK2 playing a role in leptin receptor signaling and hepatic gluconeogenesis (55). In clinical studies, baricitinib has demonstrated modest but statistically significant reductions in fasting glucose and HOMA-IR (homeostatic model assessment of insulin resistance) in RA patients with baseline insulin resistance (56). These findings suggest a potential metabolic normalization effect as inflammation subsides, and immune-metabolic pathways rebalance.

However, these effects may be agent-specific and context-dependent. For example, in psoriasis patients, upadacitinib has not consistently demonstrated improvements in metabolic biomarkers, possibly reflecting disease-specific immunopathology or the impact of baseline adiposity on drug distribution and receptor expression. Further research is needed to delineate whether JAK inhibition directly improves insulin action or simply mitigates inflammation-driven insulin resistance.

### Mitochondrial bioenergetics and cellular metabolism

JAK inhibition may also reduce mitochondrial ROS and restore cellular bioenergetics, oxidative phosphorylation, and the control of reactive oxygen species (ROS). Inhibition of JAK1 and JAK2 has been shown to modulate mitochondrial respiration in both immune and metabolic tissues (57). For instance, tofacitinib has been observed to reduce ROS production in monocytes and improve mitochondrial membrane potential in fibroblast-like synoviocytes from patients with RA (58). These findings point toward a restorative effect on mitochondrial function, which may underlie some of the anti-inflammatory and potentially anti-catabolic properties of these agents.

This mechanistic insight is particularly relevant in the context of “metabolic inflammation,” where dysfunctional mitochondria and altered bioenergetic flux fuel chronic immune activation. By restoring mitochondrial efficiency and dampening redox imbalance, tsDMARDs may contribute to a more favourable metabolic milieu, although this remains speculative and warrants confirmation *in vivo*. These pleiotropic effects necessitate careful metabolic monitoring, particularly in patients with existing cardiovascular risk.

### TYK2 inhibition: mechanistically selective and metabolically neutral

Given their focused inhibition of IL-12, IL-23, and type I interferon signalling, TYK2 inhibitors appear metabolically neutral in early studies. Their favourable safety profile may offer advantages in patients with metabolic comorbidities. TYK2 inhibitors, with agents like deucravacitinib and brepocitinib showing high efficacy in psoriasis and psoriatic arthritis with a favourable safety profile. TYK2 plays a central role in transducing signals from IL-12, IL-23, and type I interferons, all key drivers of psoriatic disease and systemic inflammation (59).

Preliminary data suggest that TYK2 inhibition may exert metabolically neutral or even beneficial effects, although this area remains under active investigation. Given their selective targeting and reduced JAK1/2 interference, TYK2 inhibitors might avoid some of the metabolic complications associated with broader JAK inhibition, such as lipid elevation or thrombotic risk (60). Moreover, their potential role in modulating adipose-tissue-resident immune cells and skin–adipose crosstalk presents an exciting area of translational research.

### Safety signals and metabolic trade-offs

While tsDMARDs offer convenience, oral administration, and broad cytokine suppression, they also raise safety concerns, particularly regarding weight gain, dyslipidemia, and coagulation abnormalities. However, Corrao S. underscored that the cardiovascular safety concerns associated with Janus kinase inhibitors in rheumatoid arthritis may be substantially mitigated through diligent LDL-cholesterol management, highlighting the need for a proactive, multidisciplinary approach to optimise patient outcomes (61). Post-marketing surveillance and registry data have suggested that JAK inhibitors may be associated with subtle increases in body weight and fat mass over time, particularly in patients with prior cachexia or high disease activity (62). The net impact of these changes on cardiovascular risk is still under debate, and risk-benefit analyses must be tailored to individual metabolic profiles.

### Biologic and synthetic therapies in atopic dermatitis

Atopic dermatitis (AD) is increasingly acknowledged as a systemic immune-mediated disease that extends beyond cutaneous manifestations, particularly in its moderate-to-severe forms. The association of AD with obesity, insulin resistance, and cardiometabolic comorbidities reflects the broader systemic impact of type 2 inflammation and its crosstalk with metabolic networks (63).

Dupilumab, a fully human monoclonal antibody targeting the IL-4 receptor alpha subunit (IL-4Rα), inhibits both IL-4 and IL-13 signalling and was the first biologic approved for AD. It has revolutionised the therapeutic approach to type 2 driven diseases, including asthma and chronic rhinosinusitis with nasal polyps. Although its principal action is on skin inflammation and pruritus, emerging data suggest dupilumab may also exert beneficial effects on systemic immunometabolic processes. Preclinical studies demonstrate normalisation of lipid composition and enhanced barrier integrity in keratinocytes treated with an IL-4/IL-13 blockade, suggesting a broader immunometabolic benefit (64). Clinical studies in obese patients with AD also report reductions in systemic inflammatory markers during treatment, though robust metabolic endpoints remain under investigation.

Two additional IL-13–targeted therapies—tralokinumab and lebrikizumab—have recently expanded the therapeutic arsenal for AD. Tralokinumab binds directly to IL-13, preventing its interaction with both IL-13 receptor alpha 1 and 2 (IL-13Rα1 and IL-13Rα2), while lebrikizumab selectively inhibits signaling via the IL-13Rα1/IL-4Rα heterodimer without interfering with the decoy receptor IL-13Rα2 (65, 66). These differences may have subtle effects on tissue remodelling or anti-inflammatory resolution. Clinical trials have demonstrated significant improvements in disease severity and quality of life with both agents. However, unlike broader immunomodulators, no relevant metabolic alterations have been observed to date, supporting their presumed metabolically neutral profile (66).

Janus kinase inhibitors (JAKi)—including baricitinib, upadacitinib, and abrocitinib—offer oral alternatives for patients with moderate-to-severe AD. These agents inhibit intracellular signaling pathways shared by several proinflammatory cytokines implicated in AD and metabolic regulation, such as IL-4, IL-6, and interferons. Their efficacy is robust and rapid, often leading to marked improvements in disease control. However, clinical trials and post-marketing data consistently show dose-dependent increases in serum lipids, particularly LDL and HDL cholesterol (67, 68). The long-term cardiovascular implications of these changes remain under investigation, particularly in high-risk populations. Among JAKi, upadacitinib and abrocitinib have shown earlier and more pronounced effects on lipid parameters, calling for regular lipid monitoring and individualized cardiovascular risk stratification (68).

**Table 2** summarises the metabolic profiles of the main biologic and synthetic agents approved for treating atopic dermatitis.

**Table 2:** Additional agents for atopic dermatitis (AD).

Drug class	Molecule	Approved indications	Metabolic effects
Anti-IL-4Rα/anti-IL-13	Dupilumab	AD, Asthma, *CRSwNP	Th2 inflammation modulation; potential improvement in insulin resistance
Anti-IL-13	Tralokinumab	AD	Neutral metabolic profile
Anti-IL-13	Lebrikizumab	AD	Neutral metabolic profile
JAK Inhibitor	Baricitinib	AD, RA	↑ LDL and HDL;
JAK Inhibitor	Upadacitinib	AD, RA, PsA	↑ LDL and HDL;
JAK Inhibitor	Abrocitinib	AD	↑ Total cholesterol and LDL
**TSLP blocker	Tezepelumab	Asthma (trials in AD ongoing)	Not yet approved for AD; early trials

*Chronic Rhinosinusitis with Nasal Polyps.

**Thymic Stromal Lymphopoietin.

In this evolving therapeutic landscape, metabolic profiling may emerge as a relevant tool to guide treatment decisions in AD. Biologic therapies with targeted and metabolically neutral actions may be preferred in patients with underlying dyslipidemia or insulin resistance, while oral agents with broader immunologic reach may be best reserved for individuals with a low cardiometabolic risk. This approach aligns with a growing emphasis on systemic health in dermatology and supports a more integrated, precision-based strategy for managing chronic inflammatory skin diseases.

### Comparative overview: metabolic fingerprints of biologic and synthetic agents

As biologic and targeted synthetic DMARDs (bDMARDs and tsDMARDs) continue to redefine therapeutic paradigms in chronic inflammatory diseases, clinicians are increasingly faced with the challenge of tailoring treatment not only based on immunologic profiles and clinical response but also on metabolic context. Given the shared inflammatory underpinnings of metabolic and rheumatologic/dermatologic diseases, understanding how each class of immunomodulatory agent influences metabolic pathways is becoming essential for precision medicine. This section aims to offer a comparative synthesis of the metabolic fingerprints associated with different DMARD classes, highlighting both favourable and potentially deleterious effects.

### Diverging lipid signatures

One of the most well-characterized metabolic effects of anti-inflammatory therapies is their impact on lipid metabolism. TNF-α inhibitors have shown inconsistent results: while some studies report improved lipid profiles with reduced atherogenic indices, others highlight elevations in total cholesterol and triglycerides without corresponding reductions in cardiovascular events (69). IL-6 inhibitors like tocilizumab, on the other hand, consistently raise both LDL and HDL levels sometimes by 20–30% but may simultaneously reduce inflammation-related cardiovascular risk by improving endothelial function and reducing CRP levels (70).

JAK inhibitors represent a distinct category. The class effect includes elevations in total cholesterol, LDL, and HDL, usually within the first 4–6 weeks of therapy. This shift is believed to reflect restoration of lipid synthesis suppressed during chronic inflammation. Importantly, while the LDL increase is numerically significant, it does not appear to alter the LDL/HDL ratio substantially (71). TYK2 inhibitors, due to their selective mechanism, may have a metabolically neutral lipid profile, although long-term cardiovascular outcomes remain to be elucidated (72).

### Weight and body composition: subtle but relevant

Weight gain and changes in body composition are increasingly recognised as clinically relevant, especially in patients with psoriatic disease or RA, who often enter treatment with preexisting obesity or sarcopenia. TNF inhibitors have been associated with mild increases in body weight and fat mass, particularly central adiposity, though lean mass may also improve, especially in cachectic patients (73). IL-17 and IL-23 inhibitors have not been robustly linked to significant weight gain, but early signals suggest possible improvement in adipose tissue inflammation and functionality (74).

Conversely, JAK inhibitors, notably tofacitinib and baricitinib, have been associated with slight body weight and fat percentage increases over time (75). The aetiology of this change remains uncertain but may involve altered leptin signalling, appetite regulation, or normalisation of catabolic states. Whether these shifts have net beneficial or harmful consequences on cardiometabolic health is currently debated.

### Comparative data from network meta-analyses

Network meta-analyses provide indirect but valuable evidence regarding the relative metabolic impact of different agents. For instance, Kristensen et al. demonstrated that while most bDMARDs improved systemic inflammation and CRP levels, only IL-6 inhibitors showed consistent effects on metabolic markers such as HbA1c and fasting glucose (76). Another comparative analysis by Singh et al. found no significant difference in major cardiovascular event rates among TNF inhibitors, IL-6 inhibitors, or JAK inhibitors. Still, it stressed the need for long-term cardiovascular outcome studies, especially in high-risk populations (77).

These findings reinforce the importance of stratifying patients by disease phenotype and metabolic endotype such as insulin-resistant, lipodystrophic, or proatherogenic profiles which may respond differently to specific immunomodulators.

### Toward therapeutic stratification based on metabolic profiles

Incorporating metabolic criteria into therapeutic decision-making is not yet standard practice but represents an evolving frontier in dermatology and rheumatology. For instance, patients with high baseline triglycerides or central obesity may benefit from agents with minimal lipid elevation (e.g., IL-17 or TYK2 inhibitors). At the same time, those with active insulin resistance might derive more benefit from IL-6 blockade. Similarly, individuals with inflammatory cachexia or muscle wasting may tolerate the anabolic effects of TNF or JAK inhibitors more favourably.

Moreover, emerging “multi-omic” approaches combining transcriptomic, metabolomic, and lipidomic data are beginning to uncover biological signatures that predict both therapeutic response and metabolic modulation. In this context, a drug’s metabolic fingerprint becomes not a side effect but a potential biomarker of efficacy and systemic benefit.

### Clinical relevance and precision medicine applications

The convergence of inflammatory and metabolic pathways in chronic immune-mediated diseases invites a paradigm shift in how clinicians approach patient stratification and therapy selection. Historically, treatment decisions in dermatology and rheumatology have been driven by clinical severity, organ involvement, and safety profiles. However, as evidence accumulates that biological and tsDMARD therapies exert metabolic effects beneficial or adverse it becomes clear that metabolic status must be integrated into the therapeutic decision-making process. This has substantial implications for both individual patient outcomes and long-term public health strategies, particularly given the global epidemic of obesity, insulin resistance, and cardiometabolic disease.

### Biologics in obese and insulin-resistant patients: rethinking standard choices

Obesity is a well-recognized modifier of disease expression and treatment response in both psoriasis and rheumatoid arthritis. Patients with high body mass index (BMI) frequently exhibit reduced efficacy to TNF inhibitors, potentially due to altered pharmacokinetics, increased drug clearance, and TNF-rich adipose tissue (78). In contrast, IL-17 and IL-23 inhibitors appear less affected by BMI, offering more consistent skin clearance and joint response across weight strata (79). This observation suggests that in obese patients, therapies targeting Th17-driven inflammation may be metabolically and immunologically more effective than TNF blockade.

Moreover, IL-6 inhibitors or specific JAK inhibitors in patients with insulin resistance or early type 2 diabetes may have dual benefits: disease control and improvement in glycemic parameters, as demonstrated in small clinical cohorts (80). These agents may be particularly attractive in patients with metabolic syndrome, where systemic inflammation and metabolic dysregulation are tightly coupled.

### Inflammometabolism as a therapeutic target

The term “inflammometabolism” captures the pathological synergy between immune activation and metabolic dysfunction. In diseases like psoriasis and PsA, where both pathways are intimately intertwined, treating inflammation alone may not suffice. A subset of patients continues to exhibit residual cardiovascular risk or progressive NAFLD despite clinical remission of the skin or joint symptoms (81). This suggests that targeting the immunometabolic axis, rather than isolated cytokines, may yield more profound systemic benefits.

Emerging strategies in this direction include combining immunomodulatory drugs with metabolic agents such as GLP-1 receptor agonists or SGLT2 inhibitors. Preliminary data indicate additive effects on inflammatory markers, body composition, and insulin sensitivity (71). Although not yet approved for this indication, such combinations represent an exciting frontier, particularly in patients with difficult-to-treat disease and concomitant metabolic comorbidities.

### Personalised medicine: moving beyond “one-size-fits-all”

Precision medicine in rheumatology and dermatology has historically focused on immune phenotyping e.g., seropositive vs. seronegative RA or plaque vs. pustular psoriasis. However, integrating metabolic endotypes into clinical algorithms offers a more comprehensive view of disease biology and therapeutic needs. For example, patients with high leptin/adiponectin ratios, elevated HOMA-IR, or features of lipotoxicity may benefit from therapies that modulate inflammation and metabolic stress, such as IL-6 or TYK2 inhibitors (82).

Advances in multi-omics technologies including transcriptomics, lipidomics, and metabolomics are making this vision increasingly attainable. Recent studies have identified metabolic biosignatures that predict treatment response to JAK inhibitors or IL-23 blockers, offering a glimpse into the future of personalised immunometabolic therapy (83). These signatures, if validated, could be incorporated into clinical practice as tools for therapeutic stratification and long-term risk reduction.

### Systemic implications and broader health outcomes

Beyond individual disease control, the metabolic consequences of immunomodulatory therapy may influence public health trajectories, particularly in patients with high cardiovascular or hepatic risk. If biologic or tsDMARD therapy contributes to improvements in endothelial function, insulin sensitivity, or hepatic steatosis, this could translate into reductions in morbidity and healthcare utilisation over time. Conversely, neglecting metabolic factors in drug selection may perpetuate silent risk accrual despite clinical remission.

This underscores the importance of integrated care models, where dermatologists, rheumatologists, endocrinologists, and cardiologists work collaboratively to identify at-risk patients, monitor metabolic parameters, and tailor therapies accordingly. In this evolving framework, inflammation is no longer treated in isolation but as part of a complex systemic network where immune and metabolic homeostasis must be addressed in concert.

### Emerging frontiers

As the conceptual framework of immunometabolism matures, new investigative horizons reshape our understanding of how biological and tsDMARD therapies interface with systemic homeostasis beyond inflammation control and metabolic modulation. Emerging tools ranging from single cell sequencing to metabolomic profiling unlock previously unrecognised therapeutic impact dimensions. These frontiers transcend traditional pharmacology, offering a systems biology perspective that may transform how immune-mediated diseases are managed, stratified, and prevented.

### Single-cell technologies and tissue-level resolution

The immune system and metabolic networks comprise highly heterogeneous cell populations with unique phenotypes and metabolic signatures. Advances in single-cell RNA sequencing (scRNA-seq) and mass cytometry (CyTOF) have enabled the dissection of these populations at unprecedented resolution. In inflamed synovium or psoriatic skin, for example, single-cell profiling has revealed distinct subpopulations of T cells, macrophages, and fibroblasts with disease- and drug-specific metabolic states (84).

These insights have direct implications for therapy. For instance, IL-17 blockade has been shown to reprogram synovial fibroblasts toward a less glycolytic and more quiescent phenotype, potentially limiting joint damage in psoriatic arthritis (85). Similarly, scRNA-seq data suggest that JAK inhibitors reduce mitochondrial stress and inflammatory gene expression in keratinocytes and monocyte-derived dendritic cells. This may underlie their broad tissue efficacy in dermatologic disease (86).

Integrating these cellular atlases with clinical phenotypes will enable the identification of responders vs. non-responders, guiding precision immunotherapy beyond clinical heuristics.

### Multi-omics integration and predictive modeling

While genomics and transcriptomics have already enriched our mechanistic understanding of immune-mediated diseases, the integration of proteomics, metabolomics, lipidomics, and microbiomics offers a more holistic view of the immunometabolic landscape. The potential of machine learning algorithms to combine these data layers and create predictive models of treatment response, metabolic risk, and disease progression is a reason for optimism in the field of immunometabolic therapies.

One promising application is the identification of metabolomic biosignatures that correlate with anti-TNF resistance or IL-23 blockade efficacy. In one study, changes in circulating branched-chain amino acids and specific phospholipid species predicted poor response to anti-TNF therapy in RA patients, regardless of clinical severity or serology (87). Likewise, psoriasis patients with high levels of proatherogenic lipid moieties responded better to TYK2 inhibition than to IL-17 inhibitors, suggesting metabolic endotyping may outperform immunologic profiling alone (88). These tools may also help identify patients who would benefit from combined metabolic-immunologic therapy, such as pairing anti-inflammatory agents with GLP-1 receptor agonists, SGLT2 inhibitors, or metformin (89).

### Therapeutic synergies: immunomodulation meets metabolism

The concept of therapeutic synergy between immunomodulatory and metabolic agents is gaining ground, offering hope for the future. Drugs like GLP-1 receptor agonists and SGLT2 inhibitors, traditionally used in endocrinology, possess anti-inflammatory properties through modulation of macrophage polarisation, adipokine secretion, and oxidative stress. Pilot trials are now exploring the use of these agents in combination with biologics in psoriasis and PsA patients with obesity or type 2 diabetes, opening up new possibilities for treatment.

The idea is to treat comorbidities in parallel and reshape the disease trajectory by concurrently modulating two interdependent systems. This strategy may also reduce required biological dosing, enhance tissue-level resolution of inflammation, and minimise off-target metabolic effects.

### Immunometabolic reprogramming: the next therapeutic era

Ultimately, the most ambitious frontier lies in directly reprogramming immune cell metabolism to restore immune tolerance and tissue homeostasis. Preclinical models have demonstrated that manipulating metabolic checkpoints such as mTOR, AMPK, or HIF-1α pathways can selectively inhibit pathogenic T cell subsets while preserving regulatory populations (90). This metabolic editing would represent a next-generation class of immunometabolic therapies if translatable to human disease.

Moreover, novel targets such as NAD+ biosynthesis, succinate dehydrogenase activity, and mitochondrial DNA stress are being explored as levers to recalibrate both immunity and metabolism in chronic inflammation (91). These efforts are still in the early phases, but the theoretical potential is transformative: a shift from immunosuppression to immune recalibration rooted in metabolic control.

### Clinical and translational implications

The interplay between immunity and metabolism is no longer an emerging curiosity; it is a foundational element of chronic inflammatory diseases and a pivotal determinant of therapeutic outcomes (92–94). As the evidence presented throughout this work illustrates, biologic and tsDMARD therapies do not simply extinguish inflammation; they actively reshape the metabolic architecture of immune, adipose, hepatic, and vascular systems. Understanding these effects is not just important, it is essential for the evolution of clinical practice. The distinct metabolic fingerprints of DMARD classes provide an opportunity to integrate metabolic status into therapeutic decision-making. Obesity, insulin resistance, visceral adiposity, and dyslipidemia should not be viewed as comorbidities but as determinants of immune phenotype and treatment response. For example, IL-17/23 inhibitors may be advantageous in patients with obesity-driven Th17 amplification, whereas IL-6 blockade or JAK inhibitors may offer dual benefits in insulin-resistant individuals.

While once aspirational, treating beyond inflammation now defines the next frontier of therapeutic reasoning. Patients with psoriasis, rheumatoid arthritis, psoriatic arthritis, or spondyloarthritis frequently carry silent burdens: insulin resistance, visceral adiposity, low-grade systemic inflammation, and heightened cardiovascular risk (95, 96). Addressing articular or cutaneous symptoms without acknowledging this broader context may yield incomplete remission and unfulfilled therapeutic potential.

Biologic agents differ in their metabolic fingerprints. TNF-α blockers offer relief for inflammatory symptoms but show mixed metabolic consequences, particularly in patients with obesity or dyslipidemia. IL-6 and JAK inhibitors demonstrate more consistent improvements in insulin sensitivity but carry lipid-related caveats. IL-17 and IL-23 blockers, while primarily targeting cutaneous or entheseal inflammation, may improve adipose-tissue immune tone. TYK2 inhibition is a promising newcomer with a potentially favourable systemic footprint (93, 96). Each of these agents operates at the intersection of immune recalibration and metabolic adaptation some more visibly than others.

In parallel, targeted synthetic DMARDs act on cytokine signalling, mitochondrial homeostasis, energy flux, and cellular stress responses (96). Their oral route, broader target range, and intracellular mechanism of action place them at a unique pharmacology and systems biology crossroads. Yet their potential cardiovascular risks demand refined patient selection and ongoing monitoring (92, 97).

The path forward is not merely therapeutic but translational. Clinical practice must now evolve to include metabolic phenotyping in treatment algorithms. Patients should be stratified by inflammatory burden, insulin sensitivity, lipid profiles, adipokine signatures, and even mitochondrial function. The incorporation of metabolic biomarkers such as adipokine profiles, HOMA-IR, lipidomics, and mitochondrial stress signatures may support true precision immunometabolic medicine. Multi-omics approaches increasingly reveal biosignatures that predict treatment response across drug classes (93, 96).

Moreover, this expanded view enables therapeutic innovation. Therapeutic synergy between immunomodulators and metabolic agents (GLP-1 receptor agonists, SGLT2 inhibitors, AMPK/mTOR modulators) represents a promising frontier, particularly for patients with combined inflammatory and metabolic disease (98). Integrating immunometabolic principles into clinical algorithms may refine risk stratification, reduce residual cardiovascular risk, and guide more personalised treatment pathways (94). Rather than managing inflammation and metabolism in parallel silos, the future lies in addressing both dimensions as one disease system.

Finally, this reframing has implications beyond individual outcomes. By attenuating the immunometabolic axis, clinicians may reduce symptoms and long-term complications such as cardiovascular events, fatty liver disease, and type 2 diabetes (94, 95). In doing so, immunometabolic therapy becomes not just treatment but prevention, a shift as profound as overdue.

In conclusion, the era of “treating to benefit by modulating both inflammation and metabolism” is here. The tools are in hand. The biology is known. What remains is the determination to embrace a truly integrated and system-oriented model of care. This paradigm shift demands a profound rethinking of Internal Medicine, not as a residual generalist field but as the strategic core of clinical coordination. Internists must be empowered and recognised as the architects of complex care pathways, bridging disciplinary silos and aligning specialised interventions within a unified, patient-centred framework.
